# Isolation of gene-edited cells via knock-in of short glycophosphatidylinositol-anchored epitope tags

**DOI:** 10.1038/s41598-019-40219-z

**Published:** 2019-02-28

**Authors:** Anastasia Zotova, Alexey Pichugin, Anastasia Atemasova, Ekaterina Knyazhanskaya, Elena Lopatukhina, Nikita Mitkin, Ekhson Holmuhamedov, Marina Gottikh, Dmitry Kuprash, Alexander Filatov, Dmitriy Mazurov

**Affiliations:** 1 Cell and Gene Technology Group, Institute of Gene Biology RAS, Moscow, Russia; 2 Faculty of Biology, Lomonosov Moscow State University, Moscow, Russia; 3 NRC Institute of Immunology FMBA of Russia, Moscow, Russia; 4 Chemistry Department, Lomonosov Moscow State University, Moscow, Russia; 5 Laboratory of Intracellular Signaling in Health and Disease, Engelhardt Institute of Molecular Biology RAS, Moscow, Russia; 6 Institute of Theoretical and Experimental Biophysics RAS, Pushchino, Russia; 7 Belozersky Institute of Physico-Chemical Biology, Lomonosov Moscow State University, Moscow, Russia

## Abstract

We describe Surface Oligopeptide knock-in for Rapid Target Selection (SORTS), a novel method to select mammalian cells with precise genome modifications that does not rely on cell cloning. SORTS is designed to disrupt the target gene with an expression cassette encoding an epitope tag embedded into human glycophosphatidylinositol (GPI)-anchored protein CD52. The cassette is very short, usually less than 250 nucleotides, which simplifies donor DNA construction and facilitates transgene integration into the target locus. The chimeric protein is then expressed from the target promoter, processed and exposed on the plasma membrane where it serves as a marker for FACS sorting with tag-specific antibodies. Simultaneous use of two different epitope tags enables rapid isolation of cells with biallelic knock-ins. SORTS can be easily and reliably applied to a number of genome-editing problems such as knocking out genes encoding intracellular or secreted proteins, protein tagging and inactivation of HIV-1 provirus.

## Introduction

The adaptation of the bacterial defense system based on clustered regularly interspaced short palindromic repeats (CRISPR), associated Cas9 protein and base-pair interaction of short RNAs with the target DNA for gene editing in diverse organisms has revolutionized functional genomic studies^1,2^. The plasticity of this technology enables targeting genes with custom guide RNAs (gRNAs) for inactivation, altered expression and epigenetic modifications, both individually and in a variety of library screening formats^3^.

Gene knockout (KO) remains the most reliable application of CRISPR/Cas9 in mammalian cells where the repair of DNA double strand breaks (DSBs) predominantly occurs via error-prone non-homology end joining (NHEJ). In contrast, the DSB-induced homology directed repair (HDR) that is required for precise genome editing is quite inefficient even when the donor DNA template design is flawless. While cells with the knockout of a surface protein can be easily sorted out based on the loss of staining with specific antibodies, the isolation of cells with knockouts of genes encoding intracellular or secreted proteins is usually achieved by cell cloning which is challenging and labor-intensive. It is also prone to accumulation of pathogenic mutations produced by NHEJ mechanism at off-target loci as well as to on-target large deletions and rearrangements^4^.

**Figure 1:**
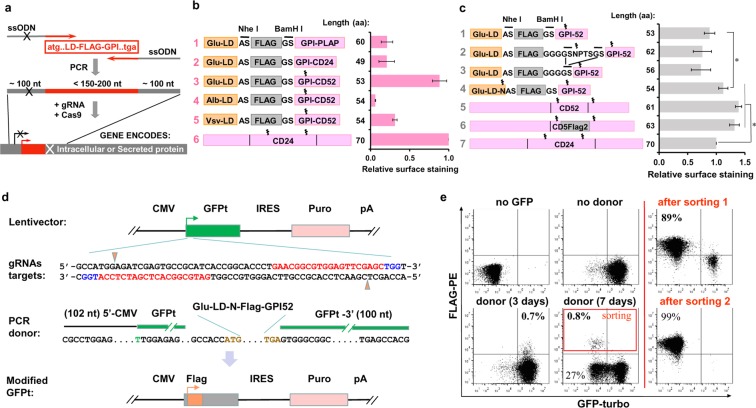
Engineering short GPI-proteins for efficient expression and knock-in selection. (**a**) Schematic representation of SORTS strategy for lentivirus transferred or single-exon genes. ssODN is a single strand oligo(deoxy)ribonucleotide. (**b,c**) Domain structures of designed GPI-proteins and bar graphs of their expression on the surface of 293 T cells transfected with the corresponding expression plasmids. Surface expression was estimated by flow cytometry as the ratio of positive to negative cells normalized to CD24 construct. Average values and standard deviations from at least three independent experiments are shown. (**d**) Design of gRNAs and PCR-donor to target *GFP-turbo* gene in bicistronic expression cassette integrated into the genome of 293 T cells by lentiviral transduction at low MOI. Target sequences and protospacer adjacent motifs (PAMs) for the paired gRNAs designed for the Cas9 nickase are highlighted in red and blue, respectively. Start and stop codons of the transgene are in brown. A to T mutation in the start codon in the 5′-arm of homology is in green. (**e**) Representative flow cytometry DotPlots showing the levels of Glu-LD-N-Flag-GPI52 KI (Y-axis) versus the levels of GFP-turbo KO (X-axis) in the presence or in the absence of donor DNA, measured at the indicated post-transfection time. The plots in the right column represent cells from the red rectangular gate sorted once or twice.

Here, we report the development of a new strategy called Surface Oligopeptide knock-in for Rapid Target Selection (SORTS) that enables the sorting of edited cells via knock-in (KI) of a short genetic element encoding an epitope targeted to the cell surface via a GPI anchor^5,6^ and designed to inactivate the start codon of the targeted gene (Fig. 1a). Its short length of 150 to 200 bp allows generation of donor DNA templates by PCR using 100 nt homology arms incorporated into synthetic primers. We show that such short donors still support a reasonable level of HDR in various CRISPR/Cas9 applications, eliminating the necessity to generate longer donor vectors by conventional cloning.

## Results

### Construction of GPI-linked tags

Each GPI-protein contains a leader sequence (LD) and a GPI-attachment signal, which are both cleaved off, whereas the middle part is GPI-anchored at its C-terminus and exported to the plasma membrane. To engineer a small GPI-protein, we selected shortest LDs from Gaussia luciferase (Glu), human albumin (Alb) and protein G of vesicular stomatitis virus (Vsv) and GPI-signals from placental alkaline phosphatase (PLAP), CD24 or CD52 used for protein export^7–9^. The domains were assembled with Flag epitope as indicated in Fig. 1b (left panel), Supplementary Table 1, and the expression levels of the resulting chimeras were compared in transiently transfected HEK 293 T cells using flow cytometry. We found that Glu-LD in combination with GPI-CD52 (# 3) provided the best level of Flag surface expression, which was comparable to that measured for native CD24 (Fig. 1b, right panel). Next, we tested whether a flexible linker between the epitope and the membrane anchor enhanced binding to the respective monoclonal antibody (mAb) and observed no positive effect (Fig. 1c, # 1–3). In contrast, addition of a signal for N-glycan attachment (Glu-LD-N-Flag-GPI52) that is known to facilitate the export of newly synthetized proteins^10^ significantly improved the level of Flag expression (Fig. 1c, #4, Supplementary Fig. 1a). Finally, we replaced six amino acids (aa) downstream of the N-glycosylation site NDT in the full-length human CD52 molecule with Flag sequence (CD5Flag2, Supplementary Fig. 1b,c) and showed that both CD5Flag2 and CD52 had better levels of surface expression than CD24 or Glu-LD-N-Flag-GPI52 (Fig. 1c, # 5–7). Of all proteins tested, Glu-LD-N-Flag-GPI52 and CD5Flag2 demonstrated the best compromise between small size and high surface expression. The microscopy examination of transfected and intracellularly stained HeLa cells confirmed that the engineered proteins were efficiently exported to the plasma membrane; the amount of chimeric protein visualized within the endoplasmic reticulum (ER) was small to moderately elevated in comparison to native CD52. (Supplementary Fig. 1d).

### Knockout of a reporter gene with GPI-epitope tag

As a proof of concept, we targeted exogenous *gfp-turbo* (*GFPt*) gene in a lentiviral construct integrated into the genome of 293 T cells transduced at low multiplicity of infection (MOI) to favor integration of a single copy per cell. The structure of the GFP lentiviral vector, sequences of the gRNAs targeting the 5′-end of *GFPt* gene with Cas9 nickase^11,12^ and the design of the donor DNA are presented in Fig. 1d and Supplementary Table 2. The donor DNA encoding GPI-Flag and containing the Kozak sequence was PCR-amplified using primers with homology arms. As shown in Fig. 1e, Flag on the cell surface was detected as early as 2–3 days post-transfection. GFPt KO cells could be detected by day 5–7, after they had lost green fluorescence due to degradation of the pre-accumulated reporter protein. Consistent with the previous reports^13–15^, the rate of NHEJ (GFP^-^ cells) greatly (~30 fold) exceeded the efficacy of HDR (Flag^+^ cells). Importantly, all Flag^+^ cells have become GFP-negative (99%) indicating high specificity of our procedure.

### KI of a GPI-epitope tag into an endogenous human locus

**Figure 2:**
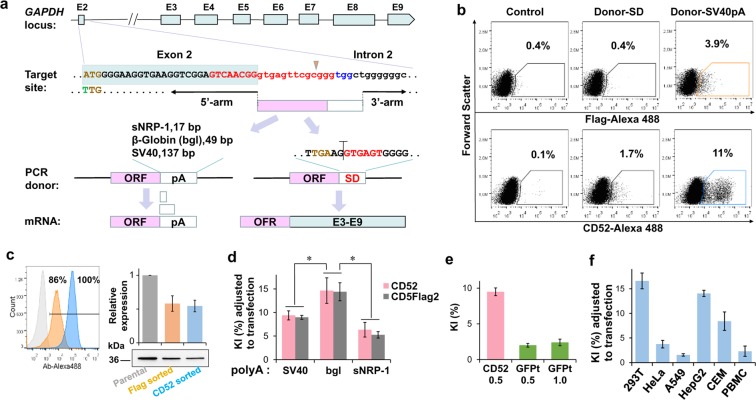
Optimization of GPI-epitope tag expression from the human *GAPDH* locus. (**a**) KI strategies tested. E1-E8 are exons, “ORF” designates either CD52 or Glu-LD-N-Flag-GPI52 coding sequence, and “pA” is a 3′-UTR containing polyadenylation signal. SD is splice donor, shown in red; see Fig. 1 for the color codes of other nucleotide sequences. (**b**) DotPlots demonstrating the efficacies of Glu-LD-N-Flag-GPI52 (upper row) or CD52 (bottom row) KI into *GAPDH* locus in 293 T cells. (**c**) Flow cytometry analysis of epitope expression by the cells gated in the right column in (**b**) and isolated by FACS-sorting (overlaid histograms on the left); levels of GAPDH expression detected by Western blot (the full-length blot is presented in Supplementary Fig. 8) and quantified using band densitometry tool (on the right). The colors of gates in (**b**) match the color codes of histograms and labels in (**c**). (**d,f**) Normalized KI rates depending on the pA (**d**), ORF (**e**) and cell type (**f**). Data from at least two (**b,c**) or three (**d–f**) independent experiments is presented as average values with standard deviations.

In comparison to vector-transferred genes, the majority of endogenous genes in somatic cells are multi-exonic and bi-allelic that makes them more challenging targets for SORTS. To test the efficiency of a GPI-construct KI into a somatic gene, we selected *GAPDH* locus, which encodes an enzyme essential for energy metabolism and therefore is permanently active and accessible to genomic nucleases. In order to minimize NHEJ-mediated inactivation of the second *GAPDH* allele that would result in cell death, we designed gRNA to target intron 2 near splice donor (SD) site. To ensure accurate splicing, polyadenylation and export of the exogenous RNA, we joined the 3′-end of the ORF (either CD52 or Glu-LD-N-Flag-GPI52) in the donor DNA either to the SD or to one of several 3′-UTR sequences containing functional polyadenylation signals (pA) (Fig. 2a). In comparison to SD, the use of the SV40 pA resulted in a much higher expression of both GPI proteins upon KI into 293 T cells (Fig. 2b). The amount of both proteins on the surface of the edited cells was sufficient to sort and grow them, albeit slowly (Fig. 2c). The low productivity of the SD construct was likely due to inefficient splicing of the modified RNA rather than nonsense-mediated RNA decoy (NMD) initiated by the premature stop-codon, since NMD inhibitors NMDI14^16^ and geneticin did not enhance the level of CD52-SD KI (Supplementary Fig. 1e). Of several 3′-UTR regions tested, a 49 bp pA from the human β-globin^17,18^ mediated transgene expression 1.5 fold stronger than the 137 bp SV40 pA while a 17 bp pA from the soluble neuropilin-1 (sNRP-1)^19^ was less effective (Fig. 2d) indicating a limit to shortening the 3′-UTR. However, the length of the ORF must be kept as short as possible, as *gfp-turbo* gene (696 bp) integrated into *GAPDH* locus ~4 fold less efficiently than *CD52* gene (183 bp) using identical homology arms (Fig. 2e). The KI rate of the optimized CD5Flag2-bglpA donor (containing the β-globin pA) tested in different human cells such as adherent carcinomas, lymphoid cells and activated peripheral blood mononuclear cells (PBMCs) varied from 1.5% to 17% (Fig. 2f), however, in all cases the population of KI cells could be clearly distinguished (Supplementary Fig. 2).

### Purification of biallelic knockouts via KI of two epitope tags

**Figure 3:**
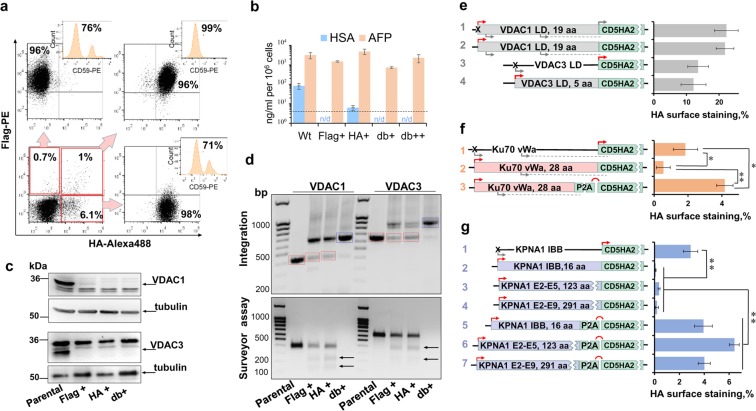
Targeting human endogenous loci for GPI-epitope tag knock-in and knockout selection. (**a**) Double KI of CD5Flag2-bglpA and CD5HA2-bglpA into the human *CD59* gene results in selection of highly pure KO cells using double positive cell sorting. 293 T cells were immunostained 3 days post transfection (lower left DotPlot) or after several rounds of Flag^+^ and/or HA^+^ cell sorting (as indicated by arrows) and analyzed for CD59 surface expression (shown on the embedded histogram plots). (**b**) ELISA quantification of HSA and AFP (control) in the supernatants of HepG2 cells targeted with two epitope tag constructs for the *HSA* gene; dashed line indicates detection limit, n/d - not detected. Flag+, HA+, db+ and db++ indicate single or double positive cell populations sorted once or twice (see Supplementary Fig. 4 for details). (**c**) WB analysis of VDAC1 (top image) and VDAC3 (bottom image) in 293 T cells after KI into respective loci and cell sorting using one or two tags (the full-length blots are presented in Supplementary Fig. 8) (**d**) PCR-amplification of targeted loci from sorted cells (top panel); DNA fragments corresponding to transgene integration generated from db+ cells (blue boxes) were excised and analyzed by Sanger sequencing, whereas DNA fragments with no integration (red boxes) were purified to detect indels using Surveyor assay (bottom gel). (**e–g**) *In-frame* strategies (left) and CD5HA2 expression (right) measured in 293 T cells after KI into indicated genes; arrows above the lane indicate active (red) or inactive (gray) start-codons *in-frame* with transgene; arrows below the lane are AUG-codons out-of-frame with CD5HA2; dashed line indicates the codon that has A/G at + 3 position and can initiate translation of short peptides; translated products as fusions of VDACs LD, Ku70 vWa (von Willebrand A-like domain) or KPNA1 IBB (Importin beta binding domain) with transgene are shown as rectangle boxes. Images in a, c, and d are representative of at least two experiments. The averaged results from three independent experiments with standard deviations are presented in b and e-g. * and **, values are different by Student’s *t-test* at p < 0.05 and p < 0.01, respectively.

To test the efficiency of the SORTS approach with regard to endogenous biallelic genes, we knocked out human surface antigen CD59, a GPI-protein itself and an inhibitor of the complement membrane attack complex. CD59 is highly expressed by many cell types and in the absence of active complement its deletion is harmless. The majority of Flag^+^ targeted 293 T cells isolated by consequent rounds of cell sorting was also CD59-negative (Supplementary Fig. 3a). However, 8% to 30% of the cells retained CD59 expression, presumably due to in-frame NHEJ-mediated repair in the second allele. In order to select cells with HDR-mediated repair in both alleles, we engineered CD5HA2-bglpA and CD5c-myc2-bglpA vectors encoding two different epitopes (Supplementary Fig. 3b and Supplementary Table 1). Interestingly, the proportion and the mean fluorescence intensity (MFI) of the cell population expressing HA epitope on the surface of transfected 293 T cells was significantly higher than with either Flag or c-myc version of the construct (Supplementary Fig. 3c). The same tendency was observed when constructs with HA and Flag epitopes were used to KI into *GAPDH* locus (Supplementary Fig. 3d). Staining the cells with different anti-tag mAbs demonstrated that the advantage of the HA tag was related to the superior level of detection by rabbit anti-HA C29F4 mAb and not to the epitope sensitivity to trypsin (Supplementary Fig. 3e). Thus, when two donor DNAs encoding HA and Flag epitopes were used at equal ratio to KI into *CD59* (Fig. 3a) and other loci (see below), in many cases both the proportion and the MFI of the HA^+^ cells exceeded those of the Flag^+^, with no negative effect on cell sorting efficiency. Single epitope sorted cells contained comparable amounts of >20% of CD59^+^ cells, whereas 99% of HA^+^ Flag^+^ double positive cells were negative for CD59 staining (Fig. 3a). To test SORTS method on genes encoding intracellular or secreted proteins, we targeted human serum albumin (HSA) in HepG2 cells and two isoforms of mitochondrial membrane proteins, VDAC1 and VDAC3 (voltage-dependent anion-selective channel) (Supplementary Table 2) in HEK 293 T cells which are not critical for cell viability^20^. As shown in Fig. 3b-c and Supplementary Fig. 4a–c, all tested genes were completely knocked out in double positive sorted cells, as well as in the majority of single epitope sorted cell populations. Thus, SORTS procedure using two different epitopes results in a highly pure population of polyclonal cells with null phenotype.

### Expression of GPI-anchored tags from endogenous start-codons

PCR analysis of the targeted VDAC1 and VDAC3 loci in the single positive cell populations produced a mixture of amplicons expected for HDR- and NHEJ-mediated repair while double positive cells predominantly gave rise to the products of HDR-mediated integration of the donor DNA (Fig. 3d, upper panel). Inactivation of the majority of the second alleles via NHEJ mechanism was confirmed by detecting indels using Surveyor-assay (Fig. 3d, bottom panel). Correct integration of the transgene was confirmed by cloning the PCR amplicons obtained from Flag^+^ HA^+^ populations and sequencing 15 clones for each of the VDAC1 and VDAC3 genes (Supplementary Figs. 5,6). We found very few sporadic mutations that localized predominantly to the sequences between homology arms and most likely resulted from Taq polymerase errors. Flag and HA sequences were detected in the amplified loci at approximately equal ratio (9 and 6 clones, respectively, for both VDAC1 and VDAC3). Interestingly, the designed mutation of the endogenous start codon in the 5′-homology arm was efficiently selected in VDAC3 clones (13/15, Supplementary Fig. 6) but was completely lost in the case of VDAC1 (0/15, Supplementary Fig. 5). Possible explanation for this discrepancy was suggested by close examination of the VDAC1 transgene design, which discovered two additional out-of-frame AUG codons (Fig. 3e). Abortive translation of short ORFs could hamper the translation of the downstream marker protein and favor translation from the VDAC1 endogenous start-codon, which was placed in-frame with the GPI-epitope tag in this particular construct. Since expression of a GPI tag fused to the N-terminal portion of the endogenous protein via in-frame KI would substantially widen options to select gRNA target sites, we further explored this idea. We made and tested several constructs with 5′- homology arms designed to fuse the CD5HA2 epitope to the N-termini of several proteins of either membrane (VDAC1, VDAC3) or cytosolic/nuclear (Ku70, KPNA1) localization (Supplementary Table 2). Short hydrophobic LD peptides from VDAC1 and VDAC3 fused to CD5HA2 (Fig. 3e, #2 and 4) did not significantly interfere with the epitope surface expression indicating that the resulting hybrid GPI-proteins were processed efficiently. However, all variants of hydrophilic N-termini from Ku70 (Fig. 3f, #2) and KPNA1 (Fig. 3g, #2–4) proteins translated in frame with the CD5HA2 strongly reduced or completely abolished the epitope expression which was restored or even enhanced by addition of the P2A ribosome skipping signal^21^ upstream of the CD5HA2 ORF (Fig. 3f, #3 and Fig. 3g, # 5–7). The P2A sequence is a universal solution that would work in all cases, however, it also increases the transgene length by more than 50 bp which should decrease the KI rate. Therefore, the optimal choice of the targeting strategy will depend on the hydrophobic properties of the target protein and on the availability of gRNA sites in the target locus.

### Depletion of essential gene products with GPI-epitope tags containing inducible degrons

**Figure 4:**
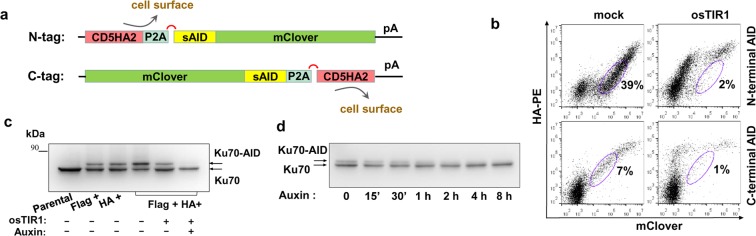
Application of GPI-epitope tags for targeting of essential genes. (**a**) Schematic illustration of constructs generated to encode for monomeric GFP mClover which is N- or C-terminally fused to smAID and CD5HA2 separated by ribosome skipping sequence P2A. (**b**) FACS plots showing 293 T cells stained for surface HA-tag. Cells were transiently cotransfected with one of the targeting plasmids and either osTIR1 expression vector or mock plasmid for 24 h and then treated with 0.5 µM auxin for 16 h. (**c**) WB analysis of native and C-terminally tagged Ku70 expressed in 293 T cells after KI and selection with one or two GPI-epitope tags. Flag^+^HA^+^ cells were also stably transduced with pUCHR-osTIR1-IRES-GFPt vector and treated with auxin for 16 h. (**d**) Dynamics of Ku70 degradation tagged with smAID. Cells (lane 5 in c) were incubated with auxin for the indicated periods of time, lysed and analyzed by WB. Images are representative of at least two experiments; the uncropped blots are shown in Supplementary Fig. 8.

Gene products that are essential for cell survival can be conditionally depleted by a number of methods at DNA, RNA or protein level^22^. In order to provide SORTS with this capability, we took advantage of an auxin-inducible degron (AID) from plant transcriptional repressor IAA17 and rice transport inhibitor response 1 (osTIR1) protein. In the presence of auxin, a non-toxic plant hormone osTIR1 associates with AID-tagged proteins and targets them for ubiquitin-dependent proteosomal degradation^23,24^. AID is the shortest known degron with fast degradation kinetics of protein that has been successfully used to tag genes via CRISPR/Cas9-mediated KI^25^. We designed a small version of AID (smAID) based on published mini-AID^26^ and other truncated variants of the degron^27,28^ and fused it to either N- or C-terminus of monomeric green fluorescent protein mClover, separating the fusion gene from the HA tag by the P2A sequence (Fig. 4a and Supplementary Fig. 7). As demonstrated in Fig. 4b, mClover was significantly degraded in the presence of osTIR1 while HA expression remained stable, suggesting that GPI-epitope was translated separately from the smAID-tagged reporter protein. Next, we added smAID at the C-terminus of human Ku70 (Supplementary Table 2), a central molecular player in DNA breaks repair, presumably critical for survival of human cells^29^. Cells sorted using two epitopes as described above for bi-allelic genes were stably transduced with osTIR1, treated with auxin and analyzed by WB (Fig. 4c). The tagged Ku70 that migrates slower than the untagged protein was detected in all sorted samples, and its level was higher in the double positive cells. Additional expression of osTIR1 in these cells slightly reduced the level of Ku70-smAID, a known phenomenon reported previously^25^. Auxin treatment completely and specifically depleted the tagged protein (Fig. 4c), with the majority of the Ku70-smAID protein disappearing during the first 2 hours of auxin exposure (Fig. 4d).

### Application of GPI-anchored tags for HIV-1 provirus inactivation

**Figure 5:**
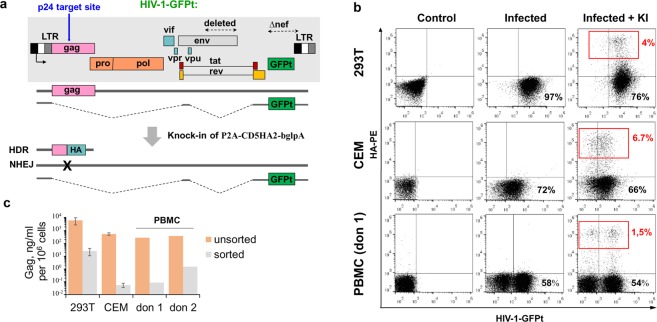
Exploring GPI-tag for selection of human cells with inactivated HIV-1. (**a**) Schematic representation of HIV-1-GFPt vector used for cell infection. For safety reasons and easy detection, viral genome was modified by deleting a portion of *env* and replacing *nef* with *gfp-turbo* gene. The transcripts for Gag and GFPt expression produced before and after KI are shown below. (**b**) Flow cytometry analysis of cells before and after HIV-1 infection and P2A-CD5HA2 KI into HIV-1 proviral genome. Four days posttransfection the indicated cells were immunostained and analyzed. (**c**) The levels of HIV-1 viral particles production in the supernatants of infected cells detected using p24 ELISA Kit prior and after FACS sorting as shown in b. Images in b and data in c obtained with 293 T and CEM cells are representative of at least two independent KI and cell sorting experiments. The results on PBMCs collected from two independent healthy donors designated as “don 1” and “don 2” (**b–c**) are shown separately.

One of the major gene editing challenges where gene modification via HDR is expected to be especially important is inactivation of HIV-1 provirus. CRISPR/Cas9 system has been applied to combat HIV^30–32^, however a significant problem was presented by escape mutants arising from error-prone NHEJ-mediated reparation of proviral DNA^33–35^. Therefore, we designed a SORTS strategy to select cells with precisely inactivated HIV-1 genome using gRNAs and P2A-CD5HA2 PCR-donors targeting a conserved region of the viral capsid protein (Fig. 5a and Supplementary Table 2). Cells (293 T, CEM or activated PBMCs) were infected with NL4–3 HIV-1-GFPt pseudotyped with VSVG Env (see Methods for details), sorted (293 T and CEM) and transfected with CRISPR/Cas9 components. As shown in Fig. 5b, an intensive expression of the HA tag from the HIV-1 *gag* provided a comfortable window to separate the edited cells which constituted from 1.5% to 7% of the total cell population, depending on the cell type. Of note, GFPt in the HIV-1 construct was translated from a fully spliced viral RNA that lacks p24 target region as schematically illustrated in Fig. 5a. Therefore, GFPt cannot be used as an indicator of HIV “cure”. Instead, we used ELISA to quantify p24 in cell supernatants, which allows assessing the degree of Gag inactivation directly. All sorted HA^+^ cells produced only residual levels of Gag that were 2 to 4 orders of magnitude lower than those in unsorted cell cultures (Fig. 5c). These data demonstrate feasibility of isolating cells with effectively “eradicated” HIV-1 using SORTS.

## Discussion

We have developed SORTS, a novel method to select cells with gene modifications that relies on HDR-mediated integration of a very short promoterless expression cassette. SORTS requires neither cell cloning nor donor vector generation and is compatible with any type of currently available^36–40^ or future programmable genomic nucleases. Isolation of polyclonal gene-edited cells by FACS is fast and may have additional advantages over clones. In particular, we have shown previously that the levels of HIV-1 and HTLV-1 replication in lymphoid cells with an expression cassette integrated into AAVS1 locus varied substantially between clones but were rather uniform in FACS-sorted polyclonal cell populations^12^. The rates of retroviral replication in VDAC1 and VDAC3 KO clones obtained in this work demonstrated similar irregularities, in contrast to SORTS-isolated polyclonal populations (data not shown).

SORTS can be applied to any gene but is especially useful for knocking out genes encoding intracellular or secreted proteins that cannot be used as markers for selection of live cells. Using two GPI-epitope tags and several different human genes we showed that KO cells can be isolated by FACS-sorting with high degree of purity. The purity of KO cells isolation using one tag, however, often varied depending on the gene (from high for VDAC3 (Fig. 3c) to much lower for CD59 (Fig. 3a)) that can be explained by non-uniform efficiency of GPI-tag integration. We believe that optimization of the target site selection and/or combination of two tags for the KI will help to further improve the effectiveness of SORTS. Additionally, the development of more efficient tools for CRISPR/Cas9 delivery and HDR enhancement can help increase the polyclonality of isolated cells without the increase of the initial sample size. This should level down the biases that can be observed with oligoclonal population of cells. In particular, the minor differences in albumin levels secreted by Flag^+^ and HA^+^ sorted cells (Fig. 3b) may be due to low efficiencies of transfection and KI followed by additional sorting procedures (Supplementary Fig. 4a), resulting in a low number of independent KI events in the isolated populations.

Inducible degradation, a standard way to assess function of essential cellular proteins, can also be integrated into SORTS, as demonstrated by introduction of an auxin-sensitive smAID tag into human Ku70, which is critically important for cell survival. Although in the latter case SORTS did not produce a pure cell population, it still significantly enriched it with the desired modification prior cloning as 5 of 14 clones (~36%) grown from the double-positive cells exclusively expressed Ku70-smAID (data not shown).

In order to further broaden gene targeting options for SORTS, we combined GPI-tag with P2A ribosome skipping sequence and developed an *in-frame* strategy to express the marker protein from endogenous start-codon. Using this modification of the technique, we were able to apply SORTS to HIV eradication. Capsid (p24) portion of *Gag* was selected for GPI-tag integration because Gag expression level is superior relative to other viral proteins. We demonstrated feasibility of HIV-1 inactivation on two human cell lines and on PBMC. Since primary cells have a restricted potency of proliferation, especially after HIV infection, experiments with PBMC did not include the step of infected (GFP^+^) cell sorting/growth used for 293 T and CEM cells. Furthermore, we had to infect PBMC with high dosage of virus in order to achieve a reasonable level of CD5HA2 KI. The high multiplicity of infection can explain why HA^+^ population of transfected PBMC contained both GFP^-^ and GFP^+^ subpopulations (Fig. 5b). We think the HA^+^GFP^-^ population represents the cells with a single copy of proviral DNA where the GPI tag with the transcription terminator was integrated. The HA^+^GFP^+^ population likely consists of cells with multiple proviral copies some of which was edited via KI enabling HA expression (Fig. 5a, HDR transcript) while any others were modified by NHEJ resulting in inactivation of Gag but not GFPt (Fig. 5a, NHEJ transcript). As quantified by ELISA, SORTS dramatically reduced the levels of HIV-1 viral particle production, though the residual level of Gag was still detectable in sorted cells. This incomplete “cure” can be explained by multiple factors such as a purity of cell sorting, a number of proviral copies integrated in individual cells, off-target integration of GPI-tag.

Further modifications of SORTS method may be aimed at the problems with epitope tag immunization or with reinfection of HIV “cured” cells. This can be done by replacing HA (Flag) with, for example, a peptide from gp41 inhibiting virus-cell fusion. As elegantly demonstrated by Matabaro *et al*.^5^, a GPI-anchored protein used for cell selection can be switched to a secreted form that in case of gp41 peptide may enhance its effectiveness by providing protection to non-edited cells. Other SORTS applications that can be envisioned include monitoring of promoter activity or inactivation of non-coding RNAs^41^.

## Methods

### Cell cultures

The human embryonic kidney 293 T cells were obtained through NIH AIDS Research and Reference Reagent Program. The human CD4 T cell line CCRF-CEM, epithelial carcinoma cell lines HeLa and A549 and hepatocellular carcinoma cell line HepG2 were purchased from ATCC. The peripheral blood mononuclear cells (PBMC) from healthy donors were isolated on the density gradient of Ficoll-Paque (Paneco, Russia), activated with 5 μg/ml of phytohemaglutinin (PHA) (Sigma-Aldrich, USA) for 3 days, and grown in the presence of 100 U/ml of recombinant human interleukin-2 (Ronkoleikin, Biotech, Russia). All experiments with the human blood samples were approved by the Human Ethics Committee of the Institute of Immunology (Moscow), and blood donors gave informed consent for the use of their samples in the described experiments. All methods were performed in accordance with relevant the guidelines and regulations. The adherent cell lines were cultured in high glucose Dulbecco’s modified Eagle’s medium (DMEM) (Sigma-Aldrich, USA) with sodium pyruvate, sodium bicarbonate, 10% fetal calf serum (FCS), 2 mM glutamine and 40 µg/ml gentamicin. CEM cells and PBMCs were maintained in RPMI 1640 medium containing 10% fetal calf serum, 2 mM glutamine and 40 µg/ml gentamicin. All transfections were performed on low passage cells; cultured cell lines were checked periodically for mycoplasma contamination.

### Plasmid construction

The detailed information about primers, cloning strategies, plasmids used for gene cloning, and Addgene depositing numbers is presented in Supplementary Table. 1. For transient expression, GPI-protein coding sequence and an epitope tag were PCR assembled and cloned into mammalian expression vector pCMVpA encoding polyA signal from SV40 late antigen. To generate expression plasmids with polyA signals derived from human soluble neuropilin-1 or β-globin, the CMV promoter with the coding sequence were merged at the 3′-end with respective polyA using PCR and cloned into pJet1.2 plasmid (Thermo Scientific, USA). For stable gene expression, the gene of interest was cloned into lentiviral vector pUCHR-IRES-GFP. The pHIV-1-GFPt vector encoding NL4-3 strain of HIV-1 with partial deletions in Env and Nef was derived from pHIG(ON) (a gift from Dr. W. S. Hu, NCI-Frederick) by subcloning *gfp-turbo* gene (Evrogen, Russia) into *Nef* region. The gRNA expressing vector pKS gRNA BB and the plasmid for the expression of wild type or nickase mutant of Cas9 (Addgene #41815) were described earlier^1,42^. All PCR DNA fragments prepared for cloning were generated using Pfu polymerase (Sibenzyme, Russia) and verified by sequencing.

### Guide RNAs and donor DNAs

Guide RNA (gRNA) protospacer sequences were selected using two web-based resources http://crispr.mit.edu/ and http://chopchop.cbu.uib.no/ and cloned into pKS gRNA BB plasmid using BbsI restriction site. The ~100 nt homology arms in a close proximity to DNA cut site were included into synthetic oligos together with ~18 nt plasmid complementarity sequences (Supplementary Table 2). Donor DNAs were PCR-amplified on a large scale using 0.4 nM of each designed long oligos (Evrogen, Russia), 10 ng of plasmid template, 25 nM dNTPs, and 10 Units of Taq DNA-polymerase (all from Sibenzyme, Russia) in a volume of 100 µl at the following settings: (95 °C-2′) × 1, (95 °C-20′′, 54 °C-20′′, 72 °C-25′′) × 15, (95 °C-20′′, 72 °C-25′′) × 30, (72 °C-5′) × 1. PCR products were run on agarose gel and gel purified using Thermo Scientific Kit.

### Transfections and infections

The HEK 293 T cells were transfected in a 12-well plate (1.5 × 10^5^ cells per well) with the Lipofectamine 2000 reagent (Thermo Scientific, USA) for 6 h according to the manufacturer’s instructions. All the other types of cells were transfected using Neon electroporation system and 100 µl Neon pipette tips (Invitrogene, USA). The following amounts of cells and electroporation settings (voltage, pulse width and numbers) were used: HepG2 5 × 10^5^–1,200 V, 50 ms × 1; A549 5 × 10^5^–1,230 V, 30 ms × 2; HeLa 5 × 10^5^–1,005 V, 35 ms × 2; CEM and activated PBMCs 10^6^–1,230 V, 40 ms × 1. To test the efficiencies of GPI-protein expression, 293 T cells were transiently transfected with 1.5 µg of expression plasmid for 2 days. For KI, 293 T cells were cotransfected with 0.75 µg of wild type or nickase spCas9 expression plasmid, 0.25 µg of gRNA expression vector and 0.5 µg of PCR-donor (0.25 µg of each donor DNA in case of using two donors). Where indicated, geneticin (Gibco) and/or NMDI14 (Sigma-Aldrich, USA) were added during transfection medium replacement. If Neon transfection was used for KI initiation, the DNAs were used in amounts of 3 µg, 1 µg, and 1 µg (0.5 + 0.5) µg, respectively. To generate lentiviral particles, 5 × 10^5^ 293 T cells in a 6 cm dish were cotransfected with 2 µg of HIV-1 packaging plasmid pCMV∆8.2 R (Addgene # 12263); 0.5 µg of the pCMV-VSV-G plasmid (Addgene # 8454) expressing protein G from vesicular stomatitis virus (VSV-G); and 3 µg of one of the transfer vector: pGIPZ (Open Biosystems, USA) for GFP-turbo stable expression (Fig. 1d,e), or pUCHR-osTIR1-IRES-GFP for smAID-mediated degradation (Fig. 4c,d). For HIV-1-GFPt infection (Fig. 5), 293 T cells were cotransfected with 5 µg of pHIV-1-GFPt and 0.5 µg of pCMV-VSV-G plasmid DNAs. Supernatants containing virus like particles (VLPs) were harvested and cleared through 0.45 µm pore size filters at 48 h posttransfection. 293 T, CEM or activated cells were infected with different doses of VLPs, and the efficiencies of transduction were determined in 2–3 days later by quantifying GFP^+^ cells. When integration of a single lentiviral copy per cell was desired, cells infected at MOI < 0.1 (<10% fluorescent cells) were selected.

### Flow cytometry and cell sorting

At indicated time post transfection, live cells in suspension or adherent cells (briefly trypsinized) were stained with respective primary and secondary antibodies using standard immunofluorescence (IF) protocol. To detect the expression of both Flag and HA epitope tags, cells were simultaneously labeled with the mouse anti-Flag clone M2 (Sigma-Aldrich, USA) and the rabbit anti-HA clone C29F4 (Cell Signaling Technology, USA) mAbs, washed with PBS, and stained with the secondary goat anti-mouse PE-conjugated and goat anti-rabbit Alexa488-labeled Abs (all from Thermo Scientific, USA). Other primary Abs used for IF included mouse anti-human CD24 clone SN3, CD59 clone MEM43 and CD52 clone HI186 (all from Exbio, Czech Republic), mouse anti-c-myc clone 9E10 (Sigma-Aldrich, USA), mouse anti-HA clone 6E2, rabbit anti-Flag clone D6W5B, rabbit anti-HA clone C29F4 PE-conjugate (all from Cell Signaling Technology, USA). The secondary goat anti-mouse or anti-rabbit Abs conjugated to PE or Alexa488 were purchased from Thermo Scientific, USA. Immunolabeling was performed in PBS containing 10% FCS and Abs diluted to final concentration of 5 µg/ml. Samples were analyzed on CytoFLEX S (Beckman Coulter, USA) flow cytometry instrument equipped with 488-nm and 561-nm lasers which were used to detect Alexa488 (or GFPt) and PE fluorescent signals, respectively. Cells were sorted using a FACSAria II Instrument (Becton Dickinson Biosciences, San Jose, CA, USA). The collected data were analyzed by CytExpert and presented using FlowJo LLC software.

### Fluorescence microscopy

HeLa cells adhered to glass coverslips in 24-well plates were transfected overnight using Lipofectamine LTX (Invitrogen, USA) in accordance to manufacturer’s protocol. Cells were washed with PBS, fixed in 4% paraformaldehyde solution (Sigma-Aldrich, USA) and permeabilized in PBS containing 0.1% saponin (Sigma-Aldrich, USA). Cells were labeled for Flag or CD52 in permeabilization solution supplemented with 2% FCS. The endoplasmic reticulum (ER) was stained with rhodamine-labeled concanavalin A (Molecular Probes, USA) diluted in serum-free PBS. Stained and washed coverslips were transferred to the slides and maintained in Dako Cytomation Fluorescent Mounting Medium. The fluorescence images were captured on Olympus IX-71 inverted epifluorescence microscope equipped with Z-axis-motorized objective revolver controlled by Olympus cellSens Dimension software via Olympus Ix2 - UCB Microscope Controller. Twenty slices in Z-stack with 0.3 µm distance in-betweens were captured and deconvolved using cellSens Dimention and Autoquant X3 software, respectively. Recorded data were processed using ImageJ software. Colocalization of GPI-proteins with the ER was estimated through Z-stacks of individual cells and from different areas of the samples as the Pearson coefficient of correlation (1.0 is full colocalization, 0 is no colocalization, and −1.0 is full exclusion).

### Western blotting

10^6^ cells were lysed in 100 µl of ice cold buffer containing 1% Triton X-100 (Sigma-Aldrich, USA), 150 mM NaCl, 10 mM Tris (pH 8.0), and a protease inhibitor cocktail (Complete Mini; Roche Applied Science). After one-hour incubation at 4 °C, insoluble material was removed by centrifugation at 14,000 × g for 10 min. Proteins in lysates were resolved by 12% SDS-PAGE under nonreducing conditions and transferred onto Immobilon PVDF membrane (GE Healthcare, USA). Western blots (WB) were probed with primary and then with horseradish peroxidase-conjugated antibodies (Cell Signaling Technology, USA). The primary Abs for WB were mouse anti- GAPDH clone 6C5 mAb (Santa Cruz Biotechnology, USA), rabbit anti-human VDAC1 Ab (Ab191440, Abcam, UK), rabbit anti-VDAC3 polyclonal Ab (Thermo Scientific, USA), and rabbit polyclonal anti-human Ku70 (Ab83501, Abcam, UK). To control protein load, blots were stained with mouse anti-human tubulin clone 12G10 mAb (Developmental Studies Hybridoma Bank at the University of Iowa). Immunoreactive bands were detected with Immobilon Western reagent (Millipore, USA) using ChemiDoc XRS molecular imager (Bio-Rad, USA).

### ELISA

To quantify the levels of the human serum albumin (HSA) and the α-fetoprotein (AFP), 3 × 10^5^ HepG2 cells in complete growth medium were plated in a 6-well plate. Next day, cells were washed twice with PBS and cultured in 2 ml of serum-free DMEM growth medium for 48 h. The levels of HSA and AFP in supernatants were measured by sandwich ELISA using commercial diluents, wash solution, blocking and detection reagents purchased from Xema-Medica Co (Russia). The 96-well ELISA plate was coated by the capture antibodies, camel anti-HSA clone KP9 (a gift from Dr. S. V. Tillib, Institute of Gene Bilogy, Moscow, Russia) or mouse anti-AFP clone AF3 mAb (Bialexa, Russia), diluted in PBS at 5 µg/ml concentration. HSA was detected with mouse anti-HSA clone HC1 mAb (Bialexa, Russia) and secondary goat anti-mouse HRP-conjugated Ab (Cell Signaling Technology, USA). AFP was detected using mouse anti-AFP clone AF7 mAb directly conjugated to HRP (Bialexa, Russia). All detection antibodies were diluted to the final concentration of 1 µg/ml. The recombinant HSA and AFP purchased from Sigma-Aldrich (USA) were used as calibration standards. The levels of HIV-1 Gag were quantified using HIV-1 p24 ELISA Kit (Zeptometrix, USA) in accordance to manufacturer’s instructions.

### Genetic analysis of targeted loci

Genomic DNA from 3 × 10^6^ parental or sorted 293 T cells was purified using Quick-gDNA MiniPrep Kit (Zymo Research, USA). The VDAC1 and VDAC3 target regions were PCR-amplified using 200 ng of genomic DNA, Pfu DNA polymerase (SibEnzyme, Russia) and primers listed in Supplementary Table 2. The DNA fragments amplified from double positive cells and containing integrated transgene were resolved on agarose gel, purified and cloned into pJet 1.2 PCR cloning vector (Thermo Scientific, USA). The DNAs from single bacterial clones were used for Sanger sequencing. The DNA fragments corresponding to target locus with no integration were purified as outlined above. Indels formation in these fragments was determined using Surveyor Mutation Kit (Transgenomic, USA) and in accordance to manufacturer’s protocols.

## Supplementary information

Supplementary Figures

Table 1

Table 2

## Data Availability

The data that support the findings of this study are available from the corresponding authors upon reasonable request; some plasmids generated in the study are available from Addgene.
