# Supplementary material for: Isolation of gene-edited cells via knock-in of short glycophosphatidylinositol-anchored epitope tags
Source: Sci Rep. 2019 Feb 28;9:3132. doi: 10.1038/s41598-019-40219-z (PMC6395743; doi:10.1038/s41598-019-40219-z)
Supplement: Supplementary file 1 — Supplementary Figures [file 41598_2019_40219_MOESM1_ESM.pdf]

SUPPLEMENTARY INFORMATION

“Isolation of gene-edited cells via knock-in of short glycophosphatidylinositol-anchored epitope tags”

Anastasia Zotova, Alexey Pichugin, Anastasia Atemasova, Ekaterina Knyazhanskaya, Elena Lopatukhina, Nikita Mitkin, Ekhson Holmuhamedov, Marina Gottikh, Dmitry Kuprash, Alexander Filatov, and Dmitriy Mazurov

a **pJet-CMV-Glu-LD-N-Flag-GPI52-bglpA**

```
ACCACCATGGGAGTCAAAGTTCTGTTTGCCCTGATCTGCATCGCTGTGGCCGAGAACGCTAGCGACTACAAAGACGATGACGACAAGGGATCCGCATCCAGCAACATAAGCGGAGGCATCTTCCTGTTCT
      M  G  V  K  V  L  F  A  L  I  C  I  A  V  A  E  N  A  S  D  Y  K  D  D  D  D  K  G  S  A  S  S  N  I  S  G  G  I  F  L  F

TCGTGGCCAATGCCATAATCCACCTCTTCTGCTTCAGTTGAATAAAATATCTTTATTTTCATTACATCTGTGTGTTGGTTTTTTGTGTG
F  V  A  N  A  I  I  H  L  F  C  F  S  *
```

b **pJet-CMV-CD5Flag2-bglpA**

```
ACCACCATGAAGCGCTTCCTCTTCCTCCTACTCACCATCAGCCTCCTGGTTATGGTACAGATACAAACTGGACTCTCAGGACAAAACGACACCGACTACAAAGACGATGACGACAAGTCAGCATCCAGCA
      M  K  R  F  L  F  L  L  L  T  I  S  L  L  V  M  V  Q  I  Q  T  G  L  S  G  Q  N  D  T  D  Y  K  D  D  D  D  K  S  A  S  S

ACATAAGCGGAGGCATTTTCCTTTTCTTCGTGGCCAATGCCATAATCCACCTCTTCTGCTTCAGTTGAATAAAATATCTTTATTTTCATTACATCTGTGTGTTGGTTTTTTGTGTG
N  I  S  G  G  I  F  L  F  F  V  A  N  A  I  I  H  L  F  C  F  S  *
```

c **pJet-CMV-CD52-bglpA**

```
ACCACCATGAAGCGCTTCCTCTTCCTCCTACTCACCATCAGCCTCCTGGTTATGGTACAGATACAAACTGGACTCTCAGGACAAAACGACACCAGCCAAACCAGCAGCCCCTCAGCATCCAGCAACATAA
      M  K  R  F  L  F  L  L  L  T  I  S  L  L  V  M  V  Q  I  Q  T  G  L  S  G  Q  N  D  T  S  Q  T  S  S  P  S  A  S  S  N  I

GCGGAGGCATTTTCCTTTTCTTCGTGGCCAATGCCATAATCCACCTCTTCTGCTTCAGTTGAATAAAATATCTTTATTTTCATTACATCTGTGTGTTGGTTTTTTGTGTG
S  G  G  I  F  L  F  F  V  A  N  A  I  I  H  L  F  C  F  S  *
```

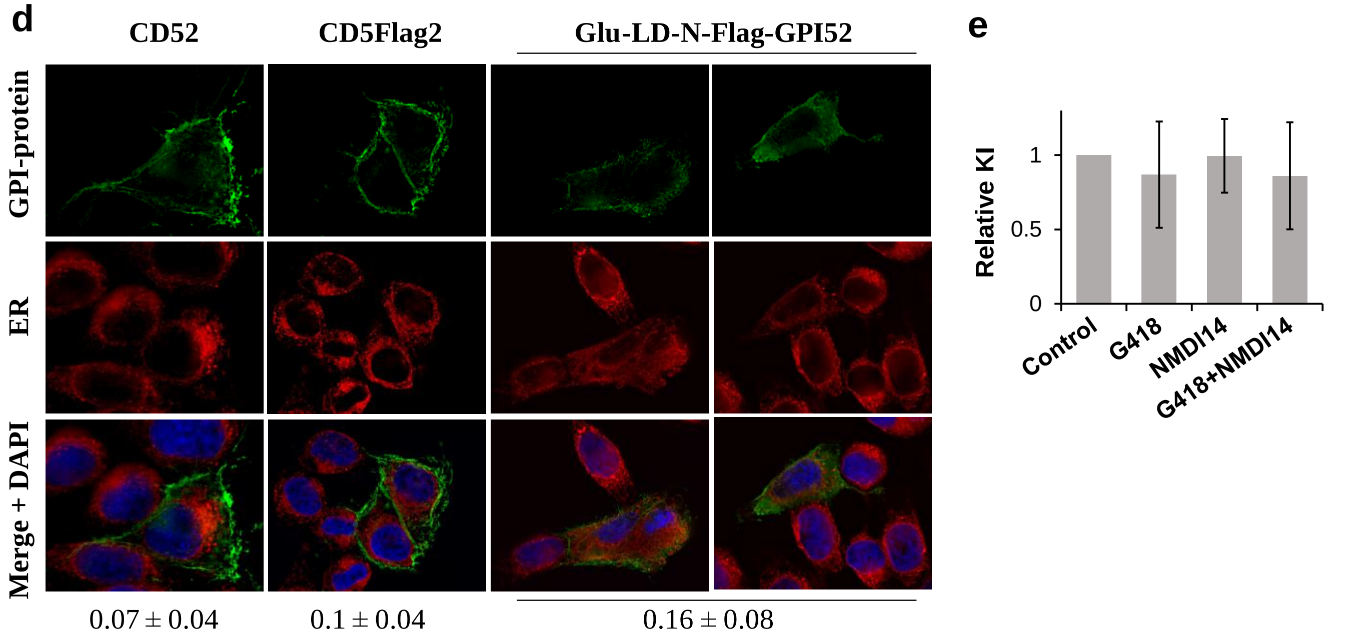

Supplementary Fig. 1. The best engineered small GPI-proteins and their expression patterns in permeabilized cells. a-c – amino acid and nucleotide sequences of coding and polyA regions of plasmids designed to express small GPI-proteins and used to generate PCR donors. Amino acids corresponding to leader peptide from *Gaussia* luciferase, Flag and human CD52 are highlighted in red, orange and blue, respectively; polyA signal from the human β-globin is underlined. **d** – representative deconvolved fluorescence microscopy images of HeLa cells transfected overnight with a construct to express one of the indicated protein demonstrate the degree of GPI-protein retention in ER (endoplasmic reticulum); ER and nucleus are stained with Rhodamin-labeled ConA and DAPI, respectively. Colocalization of stained protein with ER as a Pearson coefficients of correlation are displayed on the bottom. **e** – the levels of CD52-SD knockin into the human *GAPDH* locus in the presence of 400 μg/ml geneticin (G418) or 5 μM NMDI14 and their combination calculated relative to control (solvent) (n=3).

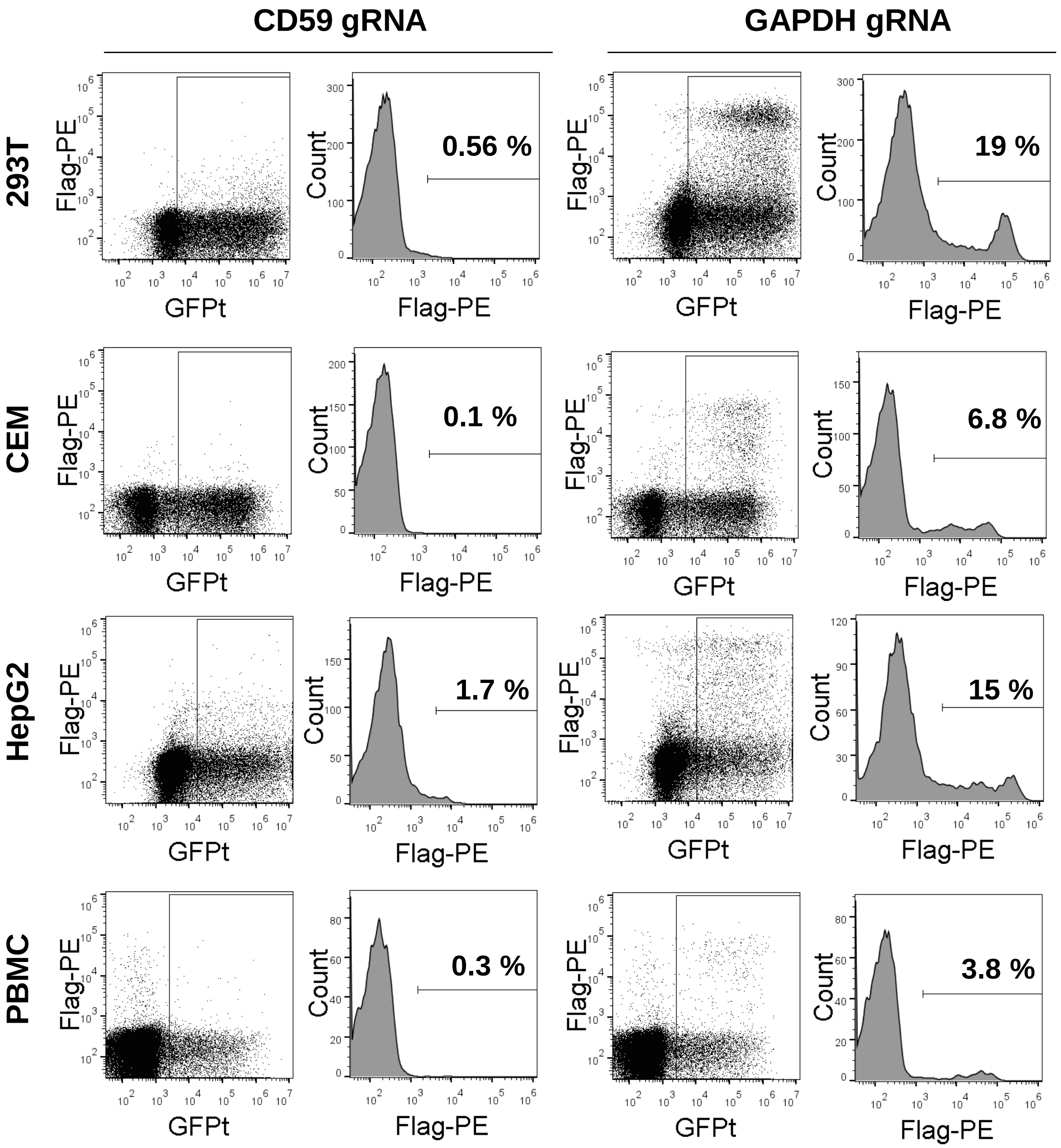

Supplementary Fig. 2. Efficiencies of CD5Flag2-bglpA knockin into *GAPDH* locus in different cell types. Indicated cells were transiently cotransfected with donor DNA, expression plasmids for gRNA, spCas9, and GFPt at a ratio 1:1:3:0.5, respectively. Three days posttransfection cells were immunostained for Flag epitope and analyzed by flow cytometry. Images are representative of three independent transfections.

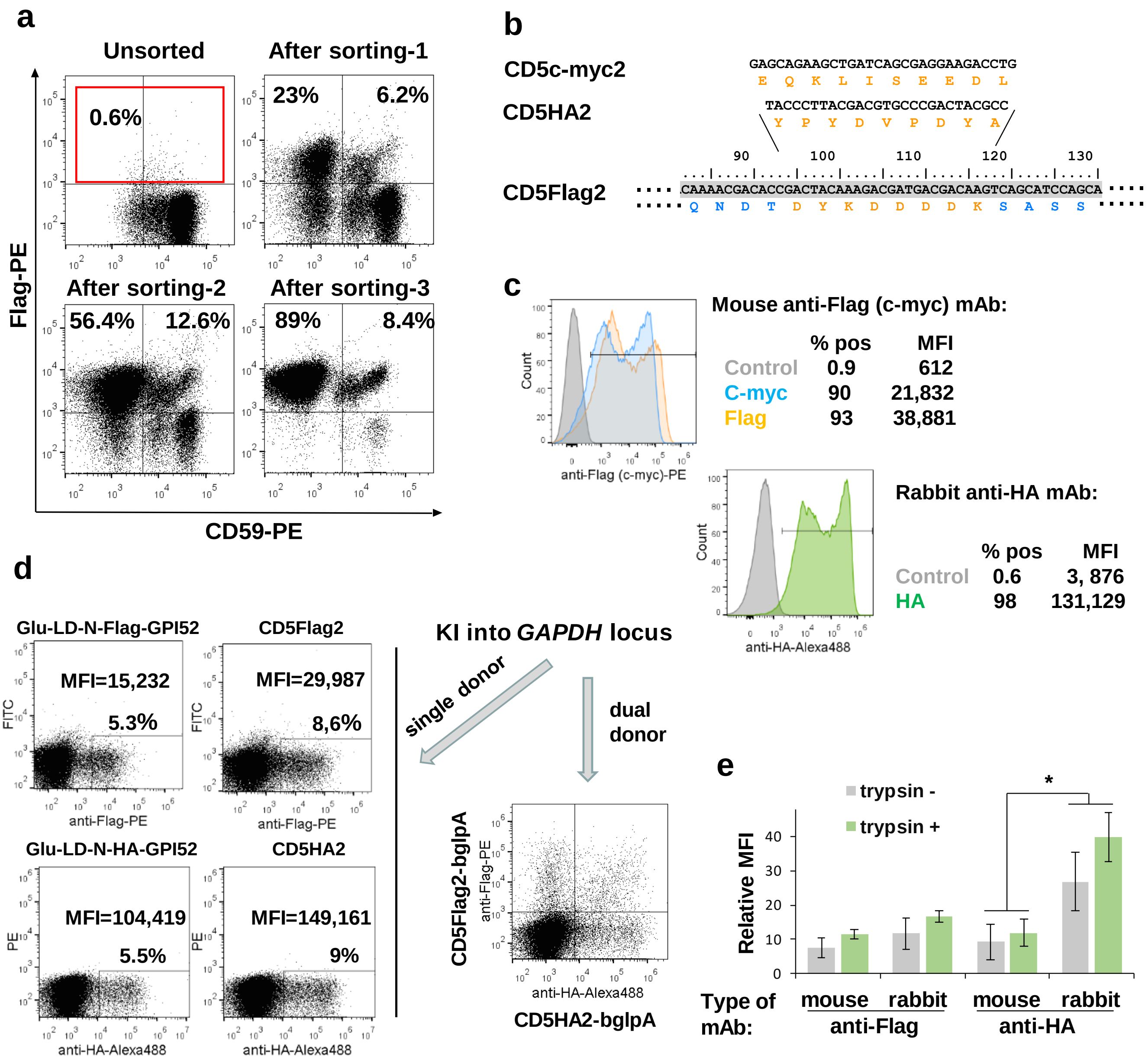

Supplementary Fig. 3. Flow cytometry analysis of Flag, HA and c-myc expression in the context of the different GPI-proteins and after labeling with different mAbs. a – Co-staining of cells with anti-Flag and anti-CD59 mAbs before and after each round of cell sorting demonstrates enrichment of population with Flag^{+} cells where a majority but not all cells are CD59^{-}. **b** – Amino acid and nucleotide sequences of HA and c-myc versions of CD52. **c** – Histograms showing the levels of the different epitope expression in the context of CD52 molecule and measured at 48 hrs posttransfection; the statistic data related to histograms and colored properly are sown on the right. **d** - DotPlots demonstrating Flag and HA epitope expression in a backbone of indicated GPI-protein constructs after KI into *GAPDH* locus. 293T cells were cotransfected with a single donor (left four images) or two donors (right image). In 3 days cells were probed with mouse anti-Flag and/or rabbit anti-HA mAb. **e** – The levels of CD5Flag and CD5HA2 cell surface staining detected with respective mAbs obtained from different sources.. Mean Fluorescence Intensities (MFI) of positively stained cells were divided by MFI levels detected for controls (no primary Ab). 293T cells in c and e were transiently transfected with respective expression plasmid and immunostained in 48 hr. Mouse Abs were anti-Flag M2, anti-myc 9E10 and anti-HA 6E2. Rabbit mAbs were anti-Flag D6W5B and anti-HA C29F4 mAb. The average results from at least three independent experiments with standard deviations are shown in e. * - the values are different at $p<0.05$

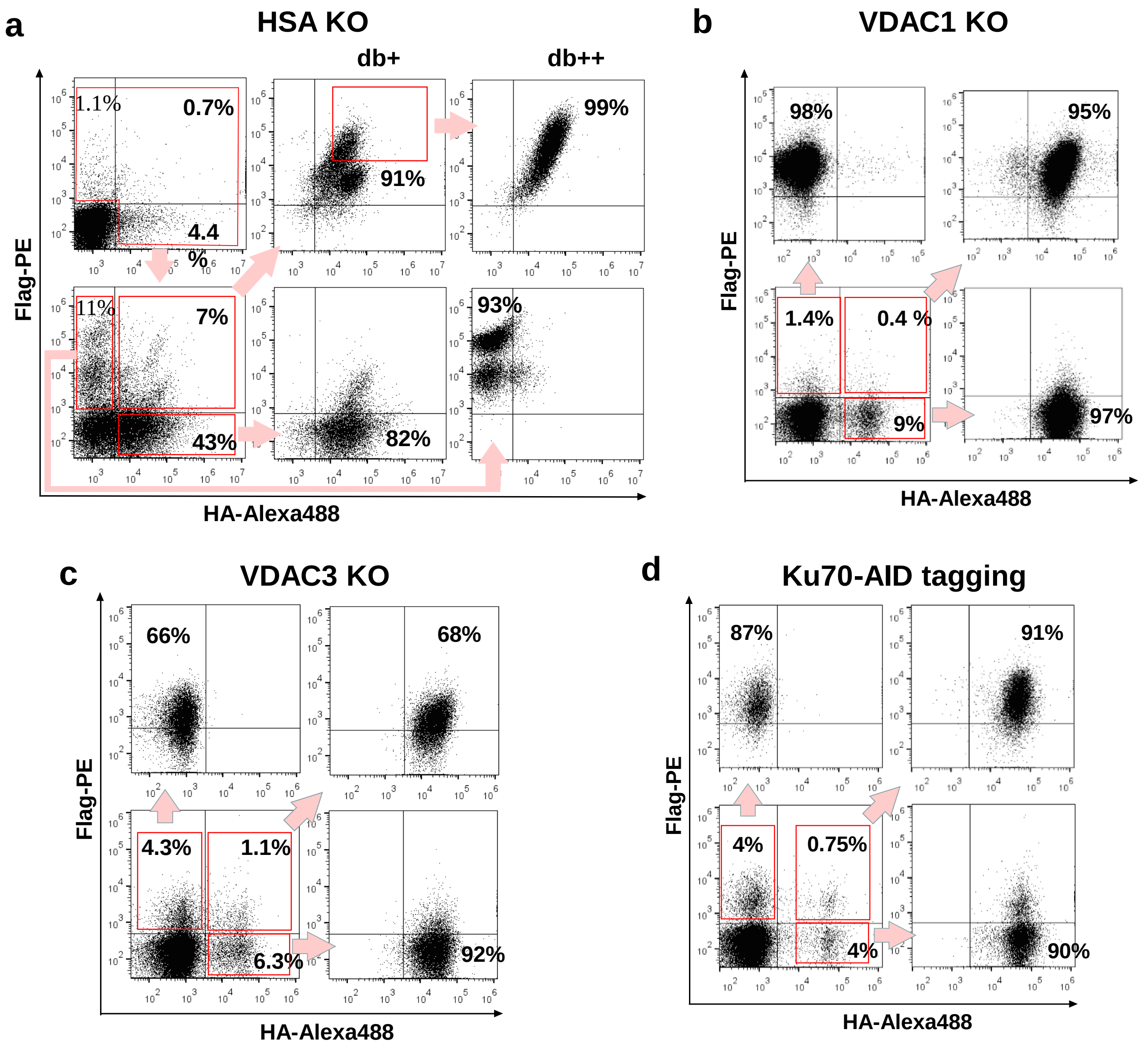

Supplementary Fig 4. FACS-sorting strategies and analysis of sorted cell populations after KI into different human loci. a-c –Flag-HA double KI strategy to isolate mono- and double positive (db+) cell populations transiently transfected to generate KI into indicated loci. **d** – Double KI approach to select cells with *Ku70* gene tagged by auxin-inducible degron (AID) at 3'-end. KI was performed into 293T (b-d) or HepG2 (a) human cells Sorting gates are highlighted in red; arrows connect these gates with corresponding cellular populations purified and analyzed after 2-3 rounds of FACS-sorting. FACS images are representative of two independent transfection/sorting experiments.

VDAC1

```
                   10        20        30        40        50        60        70        80        90       100       110       120
          ....|....|....|....|....|....|....|....|....|....|....|....|....|....|....|....|....|....|....|....|....|....|....|....|
Ref FLAG  CAATGCATTTATTCTGGTGATTCTTTTATCATAGCAGAAGTTGGCTGTGCCACCCACGTATGCCGATCTTGGCAAATCTGCCAGGGATGTCTTCACCACCATGAAGCGCTTCCTCTTCCT
Ref HA    CAATGCATTTATTCTGGTGATTCTTTTATCATAGCAGAAGTTGGCTGTGCCACCCACGTATGCCGATCTTGGCAAATCTGCCAGGGATGTCTTCACCACCATGAAGCGCTTCCTCTTCCT
1         CAATGCATTTATTCTGGTGATTCTTTTATCATAGCAGAAGATGGCTGTGCCACCCACGTATGCCGATCTTGGCAAATCTGCCAGGGATGTCTTCACCACCATGAAGCGCTTCCTCTTCCT
2         CAATGCATTTATTCTGGTGATTCTTTTATCATAGCAGAAGATGGCTGTGCCACCCACGTATGCCGATCTTGGCAAATCTGCCAGGGATGTCTTCACCACCATGAAGCGCTTCCTCTTCCT
3         CAATGCATTTATTCTGGTGATTCTTTTATCATAGCAGAAGATGGCTGTGCCACCCACGTATGCCGATCTTGGCAAATCTGCCAGGGATGTCTTCACCACCATGAAGCGCTTCCTCTTCCT
4         CAATGCATTTATTCTGGTGATTCTTTTATCATAGCAGAAGATGGCTGTGCCACCCACGTATGCCGATCTTGGCAAATCTGCCAGGGATGTCTTCACCACCATGAAGCGCTTCCTCTTCCT
5         CAATGCATTTATTCTGGTGATTCTTTTATCATAGCAGAAGATGGCTGTGCCACCCACGTATGCCGATCTTGGCAAATCTGCCAGGGATGTCTTCACCACCATGAAGCGCTTCCTCTTCCT
6         CAATGCATTTATTCTGGTGATTCTTTTATCATAGCAGAAGATGGCTGTGCCACCCACGTATGCCGATCTTGGCAAATCTGCCAGGGATGTCTTCACCACCATGAAGCGCTTCCTCTTCCT
7         CAATGCATTTATTCTGGTGATTCTTTTATCATAGCAGAAGATGGCTGTGCCACCCACGTATGCCGATCTTGGCAAATCTGCCAGGGATGTCTTCACCACCATGAAGCGCTTCCTCTTCCT
8         CAATGCATTTATTCTGGTGATTCTTTTATCATAGCAGAAGATGGCTGTGCCACCCACGTATGCCGATCTTGGCAAATCTGCCAGGGATGTCTTCACCACCATGAAGCGCTTCCTCTTCCT
9         CAATGCATTTATTCTGGTGATTCTTTTATCATAGCAGAAGATGGCTGTGCCACCCACGTATGCCGATCTTGGCAAATCTGCCAGGGATGTCTTCACCACCATGAAGCGCTTCCTCTTCCT
10        CAATGCATTTATTCTGGTGATTCTTTTATCATAGCAGAAGATGGCTGTGCCACCCACGTATGCCGATCTTGGCAAATCTGCCAGGGATGTCTTCACCACCATGAAGCGCTTCCTCTTCCT
11        CAATGCATTTATTCTGGTGATTCTTTTATCATAGCAGAAGATGGCTGTGCCACCCACGTATGCCGATCTTGGCAAATCTGCCAGGGATGTCTTCACCACCATGAAGCGCTTCCTCTTCCT
12        CAATGCATTTATTCTGGTGATTCTTTTATCATAGCAGAAGATGGCTGTGCCACCCACGTATGCCGATCTTGGCAAATCTGCCAGGGATGTCTTCACCACCATGAAGCGCTTCCTCTTCCT
13        CAATGCATTTATTCTGGTGATTCTTTTATCATAGCAGAAGATGGCTGTGCCACCCACGTATGCCGATCTTGGCAAATCTGCCAGGGATGTCTTCACCACCATGAAGCGCTTCCCCTTCCT
14        CAATGCATTTATTCTGGTGATTCTTTTATCATAGCAGAAGATGGCTGTGCCACCCACGTATGCCGATCTTGGCAAATCTGCCAGGGATGTCTTCACCACCATGAAGCGCTTCCTCTTCCT
15        CAATGCATTTATTCTGGTGATTCTTTTATCATAGCAGAAGATGGCTGTGCCACCCACGTATGCCGATCTTGGCAAATCTGCCAGGGATGTCTTCACCACCATGAAGCGCTTCCTCTTCCT

                  130       140       150       160       170       180       190       200       210       220       230       240
          ....|....|....|....|....|....|....|....|....|....|....|....|....|....|....|....|....|....|....|....|....|....|....|....|
Ref FLAG  CCTACTCACCATCAGCCTCCTGGTTATGGTACAGATACAAACTGGACTCTCAGGACAAAACGACACCGACTACAAAGACGA---TGACGACAAGTCAGCATCCAGCAACATAAGCGGAGG
Ref HA    CCTACTCACCATCAGCCTCCTGGTTATGGTACAGATACAAACTGGACTCTCAGGACAAAACGACACCTACCCTTACGACGTGCCCGACTACGCCTCAGCATCCAGCAACATAAGCGGAGG
1         CCTACCCACCATCAGCCTCCTGGTTATGGTACAGATACAAACTGGACTCTCAGGACAAAACGACACCGACTACAAAGACGA---TGACGACAAGTCAGCATCCAGCAACATAAGCGGAGG
2         CCTACTCACCATCAGCCTCCTGGTTATGGTACAGATACAAACTGGACTCTCAGGACAAAACGACACCTACCCTTACGACGTGCCCGACTACGCCTCAGCATCCAGCAACATAAGCGGAGG
3         CCTACTCACCATCAGCCTCCTGGTTATGGTACAGATACAAACTGGACTCTCAGGACAAAACAACACCGACTACAAAGACGA---TGACGACAAGTCGGCATCCAGCAACATAAGCGGAGG
4         CCTACTCACCATCAGCCTCCTGGTTATGGTACAGATACAAACTGGACTCTCAGGACAAAACGACACCTACCCTTACGACGTGCCCGACTACGCCTCAGCATCCAGCAACATAAGCGGAGG
5         CCTACTCACCATCAGCCTCCTGGTTATGGTACAGATACAAACTGGACTCTCAGGACAAAACGACACCGACTACAAAGACGA---TGACGACAAGTCAGCATCCAGCAACATAAGCGGAGG
6         CCTACTCACCATCAGCCTCCTGGTTATGGTACAGATACAAACTGGACTCTCAGGACAAAACGACACCTACCCTTACGACGTGCCCGACTACGCCTCAGCATCCAGCAACATAAGCGGAGG
7         CCTACTCACCATCAGCCTCCTGGTTATGGTACAGATACAAACTGGACTCTCAGGACAAAACGACACCGACTACAAAGACGA---TGACGACAAGTCAGCATCCAGCAACATAAGCGGAGG
8         CCTACTCACCATCAGCCTCCTGGTTATGGTACAGATACAAACTGGACTCTCAGGACAAAACGACACCGACTACAAAGACGA---TGACGACAAGTCAGCATCCAGCAACATAAGCGGAGG
9         CCTACTCACCATCAGCCTCCTGGTTATGGTACAGATACAAACTGGACTCTCAGGACAAAACGACACCTACCCTTACGACGTGCCCGACTACGCCTCAGCATCCAGCAACATAAGCGGAGG
10        CCTACTCACCATCAGCCTCCTGGTTATGGTACAGATACAAACTGGACTCTCAGAACAAAACGACACCTACCCTTACGACGTGCCCGACTACGCCTCAGCATCCAGCAACATAAGCGGAGG
11        CCTACTCACCATCAGCCTCCTGGTTATGGTACAGATACAAACTGGACTCTCAGGACAAAACGACACCTACCCTTACGACGTGCCCGACTACGCCTCAGCATCCAGCAACATAAGCGGAGG
12        CCTACTCACCATCAGCCTCCTGGTTATGGTACAGATACAAACTGGACTCTCAGGACAAAACGACACCGACTACAAAGACGA---TGACGACAAGTCAGCATCCAGCAACATAAGCGGAGG
13        CCTACTCACCATCAGCCTCCTGGTTATGGTACAGATACAAACTGGACTCTCAGGACAAAACGACACCGACTACAAAGACGA---TGACGACAAGTCAGCATCCAGCAACATAAGCGGAGG
14        CCTACTCACCATCAGCCTCCTGGTTATGGTACAGATACAAACTGGACTCTCAGGACAAAACGACACCGACTACAAAGACGA---TGACGACAAGTCAGCATCCAGCAACATAAGCGGAGG
15        CCTACTCACCATCAGCCTCCTGGTTATGGTACAGATACAAACTGGACTCTCAGGACAAAACGACACCGACTACAAAGACGA---TGACGACAAGTCAGCATCCAGCAACATAAGCGGAGG

                  250       260       270       280       290       300       310       320       330       340       350       360
          ....|....|....|....|....|....|....|....|....|....|....|....|....|....|....|....|....|....|....|....|....|....|....|....|
Ref FLAG  CATTTTCCTTTTCTTCGTGGCCAATGCCATAATCCACCTCTTCTGCTTCAGTTGAATAAAATATCTTTATTTTCATTACATCTGTGTGTTGGTTTTTTGTGTGGTGAGTGTTGCAGAGAG
Ref HA    CATTTTCCTTTTCTTCGTGGCCAATGCCATAATCCACCTCTTCTGCTTCAGTTGAATAAAATATCTTTATTTTCATTACATCTGTGTGTTGGTTTTTTGTGTGGTGAGTGTTGCAGAGAG
1         CATTTTCCTTTTCTTCGTGGCCAATGCCATAATCCACCTCTTCTGCTTCAGTTGAATAAAATATCTTTATTTTCATTACATCTGTGTGTTGGTTTTTTGTGTGGTGAGTGTTGCAGAGAG
2         CATTTTCCTTTTCTTCGTGGCCAATGCCATAATCCACCTCTTCTGCTTCAGTTGAATAAAATATCTCTATTTTCATTACATCTGTGTGTTGGTTTTTTGTGTGGTGAGTGTTGCAGAGAG
3         CATTTTCCTTTTCTTCGTGGCCAATGCCATAATCCACCTCTTCTGCTTCAGTTGAATAAAATATCTTTATTTTCATTACATCTGTGTGTTGGTTTTTTGTGTGGTGAGTGTTGCAGAGAG
4         CATTTTCCTTTTCTTCGTGGCCAATGCCATAATCCACCTCTTCTGCTTCAGTTGAATAAAATATCTTTATTTTCATTACATCTGTGTGTTGGTTTTTTGTGTGGTGAGTGTTGCAGAGAG
5         CATTTTCCATTTCTTCGTGGCCAATGCCATAATTTACCTCTTTTGCTTCAGTTGAATAAAATATCTTTATTTTCATTACATCTGTGTGTTGGTTTTTTGTGTGGTGAGTGTTGCAGAGAG
6         CATTTTCCTTTTCTTCGTGGCCAATGCCATAATCCACCTCTTCTGCTTCAGTTGAATAAAATATCTTTATTTTCATTACATCTGTGTGTTGGTTTTTTGTGTGGTGAGTGTTGCAGAGAG
7         CATTTTCCTTTTCTTCGTGGCCAATGCCATAATCCACCTCTTCTGCTTCAGTTGAATAAAATATCTTTATTTTCATTACATCTGTGTGTTGGTTTTTTGTGTGGTGAGTGTTGCAGAGAG
8         CATTTTCCTTTTCTTCGTGGCCAATGCCATAATCCACCTCTTCTGCTTCAGTTGAATAAAATATCTTTATTTTCATTACATCTGTGTGTTGGTTTTTTGTGTGGTGAGTGTTGCAGAGAG
9         CATTTTCCTTTTCTTCGTGGCCAATGCCATAATCCACCTCTTCTGCTTCAGTTGAATAAAATATCTTTATTTTCATTACATCTGTGTGTTGGTTTTTTGTGTGGTGAGTGTTGCAGAGAG
10        CATTTTCCTTTTCTTCGTGGCCAATGCCATAATCCACCTCTTCTGCTTCAGTTGAATAAAATATCTTTATTTTCATTACATCTGTGTGTTGGTTTTTTGTGTGGTGAGTGTTGCAGAGAG
11        CATTTTCCTTTTCTTCGTGGCCAATGCCATAATCCACCTCTTCTGCTTCAGTTGAATAAAATATCTTTATTTTCATTACATCTGTGTGTTGGTTTTTTGTGTGGTGAGTGTTGCAGAGAG
12        CATTTTCCTTTTCTTCGTGGCCAATGCCATAATCCACCTCTTCTGCTTCAGTTGAATAAAATATCTTTATTTTCATTACATCTGTGTGTTGGTTTTTTGTGTGGTGAGTGTTGCAGAGAG
13        CATTTTCCTTTTCTTCGTGGCCAATGCCATAATCCACCTCTTCTGCTTCAGTTGAATAAAATATCTTTATTTTCATTACATCTGTGTGTTGGTTTTTTGTGTGGTGAGTGTTGCAGAGAG
14        CATTTTCCTTTTCTTCGTGGCCAATGCCATAATCCACCTCTTCTGCTTCAGTTGAATAAAATATCTTTATTTTCACTACATCTGTGTGTTGGTTTTTTGTGTGGTGAGTGTTGCAGAGAG
15        CATTTTCCATTTCTTCGTGGCCAATGCCATAATCCACCTCTTCTGCTTCAGTTGAATAAAATATCTTTATTTTCATTACATCTGTGTGTTGGTTTTTTGTGTGGTGAGTGTTGCAGAGAG

                  370       380       390       400       410       420       430       440
          ....|....|....|....|....|....|....|....|....|....|....|....|....|....|....|....|.
Ref FLAG  GGGGTGCCTTTCAGCTCACAGACTGAGTTGGCATGTTTGAGACTTTACCGATAACTGTTTAGGAGACCTGGGTAGGTCAGC
Ref HA    GGGGTGCCTTTCAGCTCACAGACTGAGTTGGCATGTTTGAGACTTTACCGATAACTGTTTAGGAGACCTGGGTAGGTCAGC
1         GGGGTGCCTTTCAGCTCACAGACTGAGTTGGCATGTTTGAGACTTTACCGATAACTGTTTAGGAGACCTGGGTAGGTCAGC
2         GGGGTGCCTTTCAGCTCACAGACTGAGTTGGCATGTTTGAGACTTTACCGATAACTGTTTAGGAGACCTGGGTAGGTCAGC
3         GGGGTGCCTTTCAGCTCACAGACTGAGTTGGCATGTTTGAGACTTTACCGATAACTGTTTAGGAGACCTGGGTAGGTCAGC
4         GGGGTGCCTTTCAGCTCACAGACTGAGTTGGCATGTTTGAGACTTTACCGATAACTGTTTAGGAGACCTGGGTAGGTCAGC
5         GGGGTGCCTTTCAGCTCACAGACTGAGTTGGCATGTTTGAGACTTTACCGATAACTGTTTAGGAGACCTGGGTAGGTCAGC
6         GGGGTGCCTTTCAGCTCACAGACTGAGTTGGCATGTTTGAGACTTTACCGATAACTGTTTAGGAGACCTGGGTAGGTCAGC
7         GGGGTGCCTTTCAGCTCACAGACTGAGTTGGCATGTTTGAGACTTTACCGATAACTGTTTAGGAGACCTGGGTAGGTCAGC
8         GGGGTGCCTTTCAGCTCACAGACTGAGTTGGCATGTTTGAGACTTTACCGATAACTGTTTAGGAGACCTGGGTAGGTCAGC
9         GGGGTGCCTTTCAGCTCACAGACTGAGTTGGCATGTTTGAGACTTTACCGATAACTGTTTAGGAGACCTGGGTAGGTCAGC
10        GGGGTGCCTTTCAGCTCACAGACTGAGTTGGCATGTTTGAGACTTTACCGATAACTGTTTAGGAGACCTGGGTAGGTCAGC
11        GGGGTGCCTTTCAGCTCACAGACTGAGTTGGCATGTTTGAGACTTTACCGATAACTGTTTAGGAGACCTGGGTAGGTCAGC
12        GGGGTGCCTTTCAGCTCACAGACTGAGTTGGCATGTTTGAGACTTTACCGATAACTGTTTAGGAGACCTGGGTAGGTCAGC
13        GGGGTGCCTTTCAGCTCACAGACTGAGTTGGCATGTTTGAGACTTTACCGATAACTGTTTAGGAGACCTGGGTAGGTCAGC
14        GGGGTGCCTTTCAGCTCACAGACTGAGTTGGCATGTTTGAGACTTTACCGATAACTGTTTAGGAGACCTGGGTAGGTCAGC
15        GGGGTGCCTTTCAGCTCACAGACTGAGTTGGCATGTTTGAGACTTTACCGATAACTGTTTAGGAGACCTGGGTAGGTCAGC
```

- Homology arms
- Mutated endogenous start-codon and sequence mismatches
- Coding sequence
- Bgl-polyA signal
- Flag
- HA

Supplementary Fig.5. DNA sequences of CD5Flag2 and CD5HA2 PCR-donors co-integrated into VDAC1 locus. gRNA targeting region was PCR-amplified with integration-specific primers using genomic DNA isolated from sorted double positive cells (**Fig.4b**), cloned into pJet1.2 vector and analyzed by Sanger sequencing after E.coli transformation and selection of individual bacterial clones.

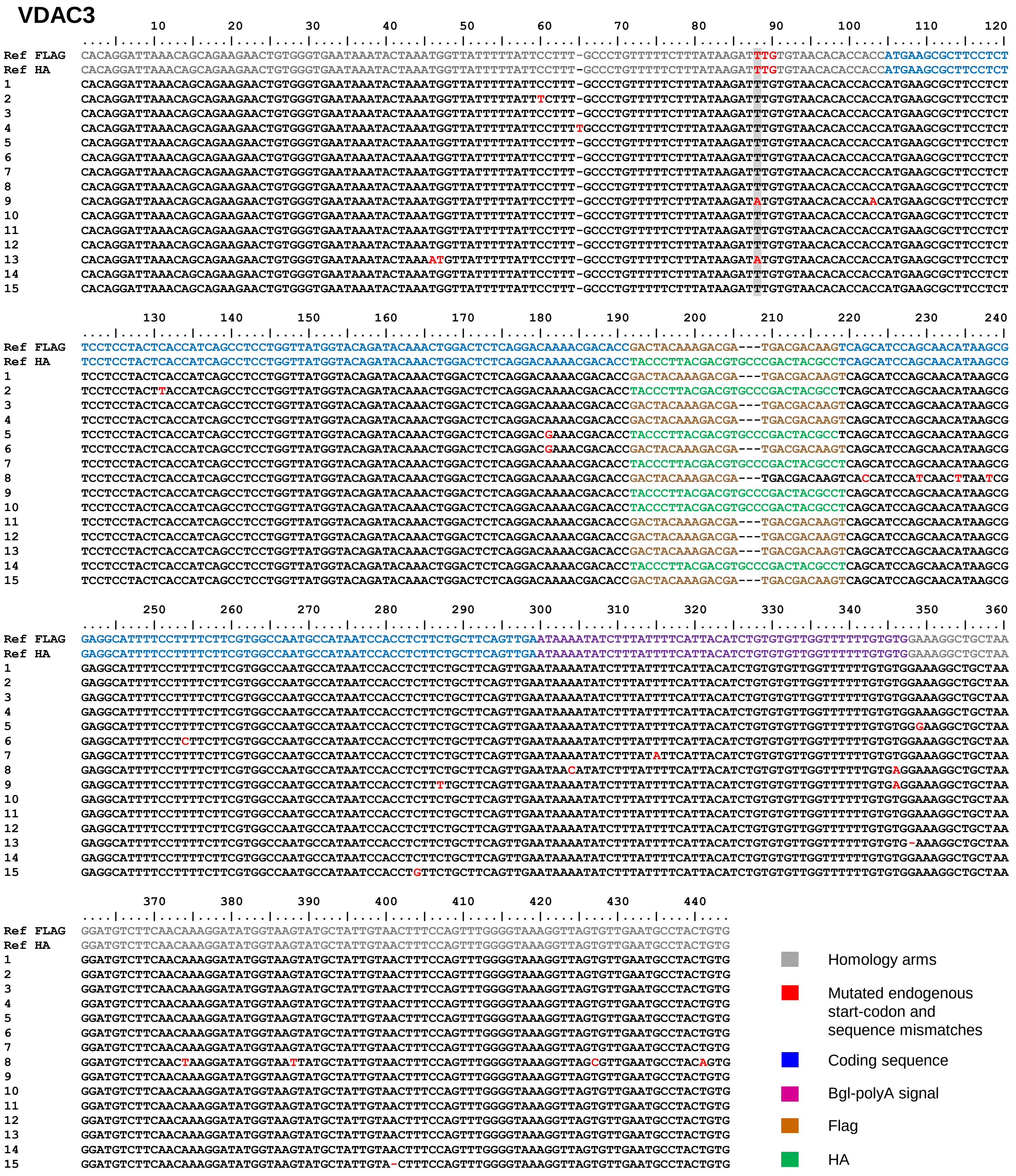

Supplementary Fig. 6. DNA sequences of CD5Flag2 and CD5HA2 PCR-donors co-integrated into VDAC3 locus. Targeted region was PCR-amplified with integration-specific primers using genomic DNA isolated from sorted double positive cells (**Fig.4c**), cloned into pJet1.2 vector and analyzed by Sanger sequencing after E.coli transformation and selection of individual bacterial clones.

pUCHR-CD5HA2-P2A-smAID-mClover

```
        10        20        30        40        50        60        70        80        90       100       110       120       130
....|....|....|....|....|....|....|....|....|....|....|....|....|....|....|....|....|....|....|....|....|....|....|....|....|....|
ACCACCATGAAGCGCTTCCTCTTCCTCCTACTCACCATCAGCCTCCTGGTTATGGTACAGATACAAACTGGACTCTCAGGACAAAACGACACCTACCCTTACGACGTGCCCGACTACGCCTCAGCATCCA
        M  K  R  F  L  F  L  L  L  T  I  S  L  L  V  M  V  Q  I  Q  T  G  L  S  G  Q  N  D  T  Y  P  Y  D  V  P  D  Y  A  S  A  S

       140       150       160       170       180       190       200       210       220       230       240       250       260
....|....|....|....|....|....|....|....|....|....|....|....|....|....|....|....|....|....|....|....|....|....|....|....|....|....|
GCAACATAAGCGGAGGCATTTTCCTTTTCTTCGTGGCCAATGCCATAATCCACCTCTTCTGCTTCAGTGGATCCGGCGCAACAAACTTCTCTCTGCTGAAACAAGCCGGAGATGTCGAAGAGAATCCTGG
S  N  I  S  G  G  I  F  L  F  F  V  A  N  A  I  I  H  L  F  C  F  S  G  S  G  A  T  N  F  S  L  L  K  Q  A  G  D  V  E  E  N  P  G

       270       280       290       300       310       320       330       340       350       360       370       380       390
....|....|....|....|....|....|....|....|....|....|....|....|....|....|....|....|....|....|....|....|....|....|....|....|....|....|
ACCGGCCAAACCTCCGGCCAAGGCACAAGTTGTGGGATGGCCACCGGTGAGATCATACCGGAAGAACGTGATGGTTTCCTGCCAAAAATCAAGCGGTGGCCCGGAGGCGGCGGCGTTCGTGAAAGTAGCT
  P  A  K  P  P  A  K  A  Q  V  V  G  W  P  P  V  R  S  Y  R  K  N  V  M  V  S  C  Q  K  S  S  G  G  P  E  A  A  A  F  V  K  V  A

       400       410       420       430       440       450       460       470       480       490       500       510       520
....|....|....|....|....|....|....|....|....|....|....|....|....|....|....|....|....|....|....|....|....|....|....|....|....|....|
AGCGTGAGCAAGGGCGAGGAGCTGTTCACCGGGGTGGTGCCCATCCTGGTCGAGCTGGACGGCGACGTAAACGGCCACAAGTTCAGCGTCCGCGGCGAGGGCGAGGGCGATGCCACCAACGGCAAGCTGA
 S  V  S  K  G  E  E  L  F  T  G  V  V  P  I  L  V  E  L  D  G  D  V  N  G  H  K  F  S  V  R  G  E  G  E  G  D  A  T  N  G  K  L

       530       540       550       560       570       580       590       600       610       620       630       640       650
....|....|....|....|....|....|....|....|....|....|....|....|....|....|....|....|....|....|....|....|....|....|....|....|....|....|
CCCTGAAGTTCATCTGCACCACCGGCAAGCTGCCCGTGCCCTGGCCCACCCTCGTGACCACCTTCGGCTACGGCGTGGCCTGCTTCAGCCGCTACCCCGACCACATGAAGCAGCACGACTTCTTCAAGTC
T  L  K  F  I  C  T  T  G  K  L  P  V  P  W  P  T  L  V  T  T  F  G  Y  G  V  A  C  F  S  R  Y  P  D  H  M  K  Q  H  D  F  F  K  S

       660       670       680       690       700       710       720       730       740       750       760       770       780
....|....|....|....|....|....|....|....|....|....|....|....|....|....|....|....|....|....|....|....|....|....|....|....|....|....|
CGCCATGCCCGAAGGCTACGTCCAGGAGCGCACCATCTCTTTCAAGGACGACGGTACCTACAAGACCCGCGCCGAGGTGAAGTTCGAGGGCGACACCCTGGTGAACCGCATCGAGCTGAAGGGCATCGAC
  A  M  P  E  G  Y  V  Q  E  R  T  I  S  F  K  D  D  G  T  Y  K  T  R  A  E  V  K  F  E  G  D  T  L  V  N  R  I  E  L  K  G  I  D

       790       800       810       820       830       840       850       860       870       880       890       900       910
....|....|....|....|....|....|....|....|....|....|....|....|....|....|....|....|....|....|....|....|....|....|....|....|....|....|
TTCAAGGAGGACGGCAACATCCTGGGGCACAAGCTGGAGTACAACTTCAACAGCCACAACGTCTATATCACGGCCGACAAGCAGAAGAACGGCATCAAGGCTAACTTCAAGATCCGCCACAACGTTGAGG
 F  K  E  D  G  N  I  L  G  H  K  L  E  Y  N  F  N  S  H  N  V  Y  I  T  A  D  K  Q  K  N  G  I  K  A  N  F  K  I  R  H  N  V  E

       920       930       940       950       960       970       980       990      1000      1010      1020      1030      1040
....|....|....|....|....|....|....|....|....|....|....|....|....|....|....|....|....|....|....|....|....|....|....|....|....|....|
ACGGCAGCGTGCAGCTCGCCGACCACTACCAGCAGAACACCCCCATCGGCGACGGCCCCGTGCTGCTGCCCGACAACCACTACCTGAGCCATCAGTCCAAGCTGAGCAAAGACCCCAACGAGAAGCGCGA
D  G  S  V  Q  L  A  D  H  Y  Q  Q  N  T  P  I  G  D  G  P  V  L  L  P  D  N  H  Y  L  S  H  Q  S  K  L  S  K  D  P  N  E  K  R  D

      1050      1060      1070      1080      1090      1100      1110
....|....|....|....|....|....|....|....|....|....|....|....|....|....|
TCACATGGTCCTGCTGGAGTTCGTGACCGCCGCCGGGATTACACATGGCATGGACGAGCTGTACAAGTAA
  H  M  V  L  L  E  F  V  T  A  A  G  I  T  H  G  M  D  E  L  Y  K  *
```

pUCHR-mClover-smAID-P2A-CD5HA2-bglpA

```
        10        20        30        40        50        60        70        80        90       100       110       120       130
....|....|....|....|....|....|....|....|....|....|....|....|....|....|....|....|....|....|....|....|....|....|....|....|....|....|
ACCACCATGGTGAGCAAGGGCGAGGAGCTGTTCACCGGGGTGGTGCCCATCCTGGTCGAGCTGGACGGCGACGTAAACGGCCACAAGTTCAGCGTCCGCGGCGAGGGCGAGGGCGATGCCACCAACGGCA
        M  V  S  K  G  E  E  L  F  T  G  V  V  P  I  L  V  E  L  D  G  D  V  N  G  H  K  F  S  V  R  G  E  G  E  G  D  A  T  N  G

       140       150       160       170       180       190       200       210       220       230       240       250       260
....|....|....|....|....|....|....|....|....|....|....|....|....|....|....|....|....|....|....|....|....|....|....|....|....|....|
AGCTGACCCTGAAGTTCATCTGCACCACCGGCAAGCTGCCCGTGCCCTGGCCCACCCTCGTGACCACCTTCGGCTACGGCGTGGCCTGCTTCAGCCGCTACCCCGACCACATGAAGCAGCACGACTTCTT
K  L  T  L  K  F  I  C  T  T  G  K  L  P  V  P  W  P  T  L  V  T  T  F  G  Y  G  V  A  C  F  S  R  Y  P  D  H  M  K  Q  H  D  F  F

       270       280       290       300       310       320       330       340       350       360       370       380       390
....|....|....|....|....|....|....|....|....|....|....|....|....|....|....|....|....|....|....|....|....|....|....|....|....|....|
CAAGTCCGCCATGCCCGAAGGCTACGTCCAGGAGCGCACCATCTCTTTCAAGGACGACGGTACCTACAAGACCCGCGCCGAGGTGAAGTTCGAGGGCGACACCCTGGTGAACCGCATCGAGCTGAAGGGC
  K  S  A  M  P  E  G  Y  V  Q  E  R  T  I  S  F  K  D  D  G  T  Y  K  T  R  A  E  V  K  F  E  G  D  T  L  V  N  R  I  E  L  K  G

       400       410       420       430       440       450       460       470       480       490       500       510       520
....|....|....|....|....|....|....|....|....|....|....|....|....|....|....|....|....|....|....|....|....|....|....|....|....|....|
ATCGACTTCAAGGAGGACGGCAACATCCTGGGGCACAAGCTGGAGTACAACTTCAACAGCCACAACGTCTATATCACGGCCGACAAGCAGAAGAACGGCATCAAGGCTAACTTCAAGATCCGCCACAACG
 I  D  F  K  E  D  G  N  I  L  G  H  K  L  E  Y  N  F  N  S  H  N  V  Y  I  T  A  D  K  Q  K  N  G  I  K  A  N  F  K  I  R  H  N

       530       540       550       560       570       580       590       600       610       620       630       640       650
....|....|....|....|....|....|....|....|....|....|....|....|....|....|....|....|....|....|....|....|....|....|....|....|....|....|
TTGAGGACGGCAGCGTGCAGCTCGCCGACCACTACCAGCAGAACACCCCCATCGGCGACGGCCCCGTGCTGCTGCCCGACAACCACTACCTGAGCCATCAGTCCAAGCTGAGCAAAGACCCCAACGAGAA
V  E  D  G  S  V  Q  L  A  D  H  Y  Q  Q  N  T  P  I  G  D  G  P  V  L  L  P  D  N  H  Y  L  S  H  Q  S  K  L  S  K  D  P  N  E  K

       660       670       680       690       700       710       720       730       740       750       760       770       780
....|....|....|....|....|....|....|....|....|....|....|....|....|....|....|....|....|....|....|....|....|....|....|....|....|....|
GCGCGATCACATGGTCCTGCTGGAGTTCGTGACCGCCGCCGGGATTACACATGGCATGGACGAGCTGTACAAGCCAGCCAAACCTCCGGCCAAGGCACAAGTTGTGGGATGGCCACCGGTGAGATCATAC
  R  D  H  M  V  L  L  E  F  V  T  A  A  G  I  T  H  G  M  D  E  L  Y  K  P  A  K  P  P  A  K  A  Q  V  V  G  W  P  P  V  R  S  Y

       790       800       810       820       830       840       850       860       870       880       890       900       910
....|....|....|....|....|....|....|....|....|....|....|....|....|....|....|....|....|....|....|....|....|....|....|....|....|....|
CGGAAGAACGTGATGGTTTCCTGCCAAAAATCAAGCGGTGGCCCGGAGGCGGCGGCGTTCGTGAAAGTAGGATCCGGCGCAACAAACTTCTCTCTGCTGAAACAAGCCGGAGATGTCGAAGAGAATCCTG
 R  K  N  V  M  V  S  C  Q  K  S  S  G  G  P  E  A  A  A  F  V  K  V  G  S  G  A  T  N  F  S  L  L  K  Q  A  G  D  V  E  E  N  P

       920       930       940       950       960       970       980       990      1000      1010      1020      1030      1040
....|....|....|....|....|....|....|....|....|....|....|....|....|....|....|....|....|....|....|....|....|....|....|....|....|....|
GACCGAAGCGCTTCCTCTTCCTCCTACTCACCATCAGCCTCCTGGTTATGGTACAGATACAAACTGGACTCTCAGGACAAAACGACACCTACCCTTACGACGTGCCCGACTACGCCTCAGCATCCAGCAA
G  P  K  R  F  L  F  L  L  L  T  I  S  L  L  V  M  V  Q  I  Q  T  G  L  S  G  Q  N  D  T  Y  P  Y  D  V  P  D  Y  A  S  A  S  S  N

      1050      1060      1070      1080      1090      1100      1110      1120      1130      1140      1150
....|....|....|....|....|....|....|....|....|....|....|....|....|....|....|....|....|....|....|....|....|....|
CATAAGCGGAGGCATTTTCCTTTTCTTCGTGGCCAATGCCATAATCCACCTCTTCTGCTTCAGTTGAATAAAATATCTTTATTTTCATTACATCTGTGTGTTGGTTTTTTGTGTG
  I  S  G  G  I  F  L  F  F  V  A  N  A  I  I  H  L  F  C  F  S  *
```

Supplementary Fig. 7. Partial sequences of plasmids generated for tagging genes with smAID using GPI-epitope tag selection. CD52 – blue, HA – orange, P2A – red, smAID – magenta, mClover – green, polyA from human β-globin is underlined. Nucleotide sequences used for PCR-donor amplification are highlighted in grey.smAID sequence was obtained by aligning the amino acid sequences of three published versions of AID (see references in the main text) and selecting the region common to all of them.

Figure 2c

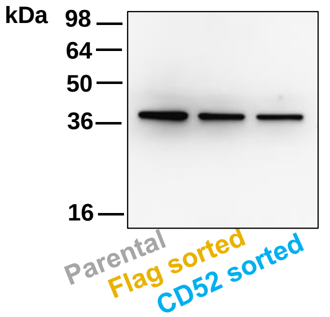

Figure 3c

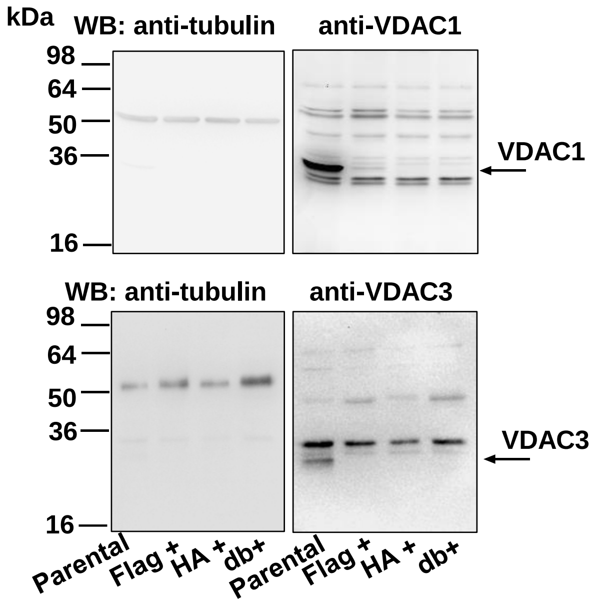

Figure 4c

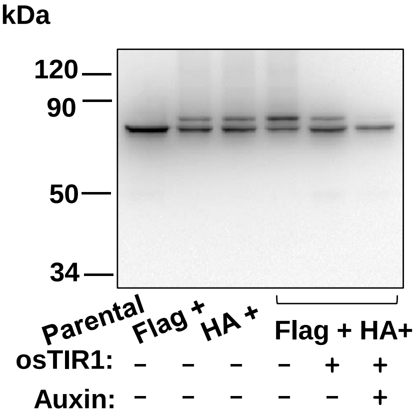

Figure 4d

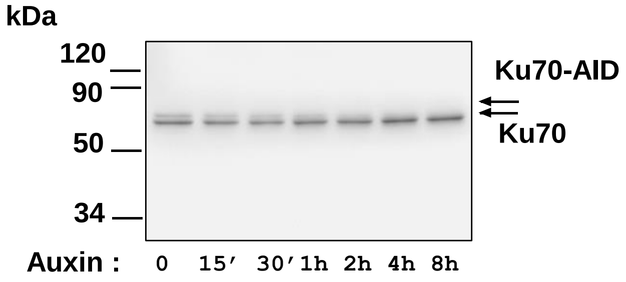

Supplementary Fig. 8. Full-length Western blot images which correspond to the cropped images presented in the main figures.
